# Expression levels of multidrug resistance-associated protein 4 (MRP4) in human leukemia and lymphoma cell lines, and the inhibitory effects of the MRP-specific inhibitor MK-571 on methotrexate distribution in rats

**DOI:** 10.3892/etm.2012.627

**Published:** 2012-06-29

**Authors:** KENJI TAKEUCHI, MASAKAZU SHIBATA, EIJI KASHIYAMA, KEN UMEHARA

**Affiliations:** Department of Drug Metabolism, Drug Safety Research Center, Tokushima Research Institute, Otsuka Pharmaceutical Co., Ltd., Kawauchi-cho, Tokushima 771-0192, Japan

**Keywords:** multidrug resistance-associated protein 4, leukemia, lymphoma, methotrexate, MK-571

## Abstract

In the development of anti-blood cancer drugs, the chronic myelocytic leukemia (KU812), acute myelocytic leukemia (KG-1) and lymphoma (U937) cell lines are commonly used in preclinical pharmacology studies as human cancer xenograft models in mice. In the present study, mRNA expression levels of typical human ATP-binding cassette (ABC) transporters in these human blood cancer cell lines were analyzed by real-time polymerase chain reaction (RT-PCR). Based on the results, the expression level of multidrug resistance-associated protein 4 (MRP4) was found to be extremely high in KU812 cells compared with those of other transporters. Additionally, MRP4 expression levels were found to be relatively high in U937, KG-1 and a blood cell line derived from a healthy subject (RPMI 1788). In addition, to elucidate the contribution of MRP4 to the methotrexate (MTX) distribution in normal blood cells and tissues, [^3^H]MTX was intravenously (i.v.) administered to two groups of rats. Animals in one group received [^3^H]MTX only; the other group was concomitantly administered i.v. MK-571, a typical inhibitor of MRP transporters. No marked difference was observed between the two groups; the Kp values (tissue concentration/plasma concentration) of the concomitant group showed slightly higher values compared with those of the MTX alone group in erythrocytes (1.4 times, P<0.001), spleen (1.3 times, P<0.05) and thymus (1.2 times, P<0.05), respectively. Although in the present study we could not evaluate the direct involvement of MRP4 in blood cancer cells in which MRP4 expression was excessively high, these results suggest a possible functional role of MRP4 in blood cancer cells and albeit only slightly in normal blood cells/tissues.

## Introduction

ATP-binding cassette (ABC) transporters, which act as efflux pumps, play a role in drug pharmacokinetics (1). It has been revealed that ABC transporters are expressed in various tissues, including the liver, kidneys, small intestine and brain (2). ABC transporters are also considered to be one of the main causes of multidrug resistance of anticancer drugs and are a major issue in the clinical setting (3).

Although numerous findings have previously been acquired with regard to the expression patterns of ABC transporters in typical tissues, including the liver, kidneys, small intestine and brain, information on the expression levels in blood cells remains inadequate (4). As for solid cancer cells, the relatively abundant expression and functional mechanisms of ABC transporters, including multidrug resistance 1 (MDR1), multi-drug resistance-associated protein 1 (MRP1) and BCRP, have been previously reported (3). In addition, the solute carrier protein (SLC) transporters (e.g. PEPT1 and OATP1B3), which play a functional role as influx pumps for drugs, are also expressed in solid cancer cells in excess (5–7). The clarification of expression levels of drug transporters in blood cancer cells would provide critical background data for developing new drugs for blood cancer, and examination of the affinity of drugs to transporters would provide useful data on multiple drug resistance.

Methotrexate (MTX) is currently used as a therapeutic drug for acute leukemia and chronic myelogenous leukemia, and is reported to be actively transported into normal and cancer cells (8). MTX is a typical substrate of MRP transporters (e.g. MRP1, 2 and 4) (9,10), and the efflux transport of MTX via MRP transporters expressed on the blood cell membrane may be functioning to a certain extent.

Although there is insufficient information on MRP4 expression in normal rat blood cells at present, the possibility of the efflux function of MRP4/5 in normal rat erythrocytes has previously been suggested (11). In addition, the contribution of MRP4 as the efflux pump in bone marrow, spleen, thymus and the gastrointestinal tract was suggested in an *in vivo* study using Mrp4-knockout mice (12). In the present study, the relative mRNA expression levels of ABC transporters in human blood cancer cell lines were measured. Secondly, MTX and an MRP inhibitor (MK-571) were coadministered to normal rats to investigate the contribution of MRP4 in normal blood cells and other tissues by measuring the MTX concentrations.

MTX is mainly excreted in the urine both in rats (13) and humans (14). Accordingly, side effects of MTX would be expected if the inhibitors of MRP transporters were concomitantly administered, which would cause inhibition of the renal clearance of MTX at the renal proximal tubule. In the present *in vivo* study using rats, to avoid the inhibitory effect against the renal clearance of MTX, MK-571 was selected as the concomitant drug possessing inhibitory potency for MRP transporters, which demonstrates a typical bile-excretion pharmacokinetic property (15). The conversion of MTX to 7-hydroxy-MTX has been reported as a main metabolic pathway of MTX in animals and humans (16,17). As the reported serum concentration of 7-hydroxy-MTX was significantly lower than that of MTX following intravenous (i.v.) bolus administration of MTX to rats (17), the influence of MTX metabolites in the present *in vivo* study may not be so large.

## Materials and methods

### RNA extraction and cDNA synthesis

Human blood cancer cell lines, KU812 (chronic myelocytic leukemia), KG-1 (acute myelocytic leukemia), U937 (lymphoma) and RPMI 1788 (normal blood cell line derived from a healthy subject), were obtained from the Health Science Research Resources Bank (Osaka, Japan). Total RNA extraction from the cells was carried out according to the manufacturer’s instructions using the RNAqueous kit (Ambion, Austin, TX, USA). First-strand cDNA synthesis was performed with the Reverse Transcription system (Roche, Mannheim, Germany). cDNA derived from healthy human liver was purchased from BioChain (Newark, CA, USA).

### Real-time polymerase chain reaction (RT-PCR)

The obtained cDNA was diluted with water and 10 μl was used for amplification. Parameter-specific primer sets optimized for the LightCycler (RAS) for the measurement of human transporters [MDR1 (ABCB1), BCRP (ABCG2), MRP1 (ABCC1), MRP2 (ABCC2), MRP3 (ABCC3), MRP4 (ABCC4), MRP5 (ABCC5), PEPT1 (SLC15A1) and OATP1B3 (SLCO1B3)], human cytochrome P450s (CYPs: CYP1A2, CYP2A6, CYP2B6, CYP2C9, CYP2C19, CYP2D6 and CYP3A4) and housekeeping genes [human glyceraldehyde 3-phosphate dehydrogenase (GAPDH) and human TATA binding protein (TBP)] were developed by and purchased from Search-LC GmbH (Heidelberg, Germany). The PCR was performed with the LightCycler FastStart DNA SYBR Green kit (RAS) according to the manufacturer’s instructions and as described previously (18). To control for the specificity of the amplification products a melting curve analysis was performed and no amplification of unspecific products was observed. The data of two independent analyses for each sample and parameter were averaged. The copy number was normalized by the two housekeeping genes, GAPDH and TBP. As the relative expression levels obtained from the two housekeeping genes were almost identical, the copy number is represented as the number of transcripts per 10^3^ copies of GAPDH.

### Materials

The ^3^H-labeled MTX ([^3^H]MTX) disodium salt [specific activity, 21.0 Ci/mmol, 1.0 mCi/23.8 μg/ml, ethanol:water (4:6) solution] was purchased from Moravek Biochemicals (Brea, CA, USA). The MK-571 sodium salt hydrate was purchased from Sigma Aldrich (Tokyo, Japan).

### Animals

Male Sprague-Dawley (SD) rats aged 6 to 7 weeks and weighing between 230 and 260 g were purchased from Charles River Japan, Inc. (Shiga, Japan) for the *in vivo* study. Animals were housed at least 7 days prior to experiments under controlled environmental conditions (23±2°C, 60±10% humidity and a 12-h light/12-h dark cycle) and fed a commercial food diet (CRF-1, Oriental Yeast, Tokyo, Japan) with water available *ad libitum*. The experimental protocols and procedures were reviewed and approved by the Animal Ethics Committee of Otsuka Pharma Ltd.

### Preparation of dosing solution

Appropriate quantities of [^3^H]MTX were diluted with water or MK-571 solution (2.5 mg/ml in water) to adjust the specific activity required for the dose preparation (0.1 mCi/kg).

### Time course of the plasma and tissue concentrations of [^3^H]MTX following i.v. bolus administration to rats

[^3^H]MTX (2.38 μg/kg, 4.76 nmol/kg) was administered i.v. as a bolus into the tail vein to two groups of rats. The animals in one group received [^3^H]MTX only, the other group was concomitantly administered i.v. MK-571 (1 mg/kg, 1.94 μmol/kg). Following drug administration, the blood was drawn from the inferior vena cava with a heparinized syringe. The sampling times for venous blood were 0.25, 1 and 4 h following administration. After blood sampling from each animal, the liver, kidneys, spleen, thymus, whole brain (cerebrum, cerebellum and medulla oblongata), bone marrow and mesenteric lymph nodes were dissected, then washed with saline and stored in a freezer (−20°C) until sample preparation.

### Sample collection and preparation

Approximately 2 ml of blood was transferred to polypropylene tubes and was rapidly centrifuged for 10 min at 1,800 × g at 4°C to obtain plasma. A portion of blood was encapsulated in a hematocrit (Ht) capillary (n=1) and centrifuged for 5 min at 10,500 × g at room temperature, and the Ht-value was measured by Ht reader. The residual blood was divided into two portions; whole blood sample (100 μl) for radioactivity measurement and the specimen for blood cell separation (remaining blood). For blood cell separation, an equivalent amount of PBS (−) (pH 7.4; Invitrogen, Carlsbad, CA, USA) was added to 2 ml of blood, and a layer (4 ml) of the diluted blood was put over 3 ml Lympholyte^®^-Mammal (Cedarlane, Burlington, NC, USA) using a large Pasteur pipette with as little agitation as possible at the interface. Following centrifugation for 20 min at 800 × g at room temperature, the cells (lymphocyte, monocyte and erythrocyte fractions) were carefully removed from the interface. The tissues (approximately 100 mg or less), with the exception of the kidneys and whole brain, were transferred directly to a glass vial and used for the preparation of radioactivity counting samples. The kidneys and whole brain were homogenized with 2 volumes of saline using a polytron homogenizer (Kinematica, Bohemia, NY, USA), and 0.1 ml of homogenates (precise weight) was used for the preparation of the radioactivity counting samples. A 1-ml portion of the tissue solubilizer (Nacalai Tesque, Kyoto, Japan) was added to each sample, with the exception of plasma, and the resulting mixture was treated with a tissue-dissolving apparatus (Biomerit, Sekisui Medical, Tokyo, Japan) for approximately 30 min. The solubilized sample was mixed with 15 ml of Hionic-Fluor (PerkinElmer, San Jose, CA, USA) for radioactivity measurement. The plasma was mixed with 1 ml of water and 15 ml of Ultima Gold (PerkinElmer) and the radioactivity was counted.

### Liquid scintillation counter analysis

The radioactivity of samples was counted using a liquid scintillation counter (Packard, St. Paul, MN, USA). The counting efficiency was corrected by the external standard method. The background value (dpm) was determined by measuring the scintillation cocktail alone.

### Distribution ratio to erythrocytes

The blood cell distribution ratio (%) was calculated from the radioactivity concentrations in the blood (Cb) and plasma (Cp) as well as the hematocrit value (Ht) using the following equation: Blood cell distribution ratio (%) = [1 − Cp/Cb × {(100 − Ht)/100}] × 100.

### Statistical analysis

In the *in vivo* study using rats, data are expressed as mean values ± SD. The comparison between the MTX alone group and the MTX and MK-571 concomitant group for every time point was carried out by Student’s t-test. P<0.05 was considered to indicate a statistically significant result.

## Results

### mRNA expression level of typical drug transporters in human blood cancer cell lines

**Figure 1:**
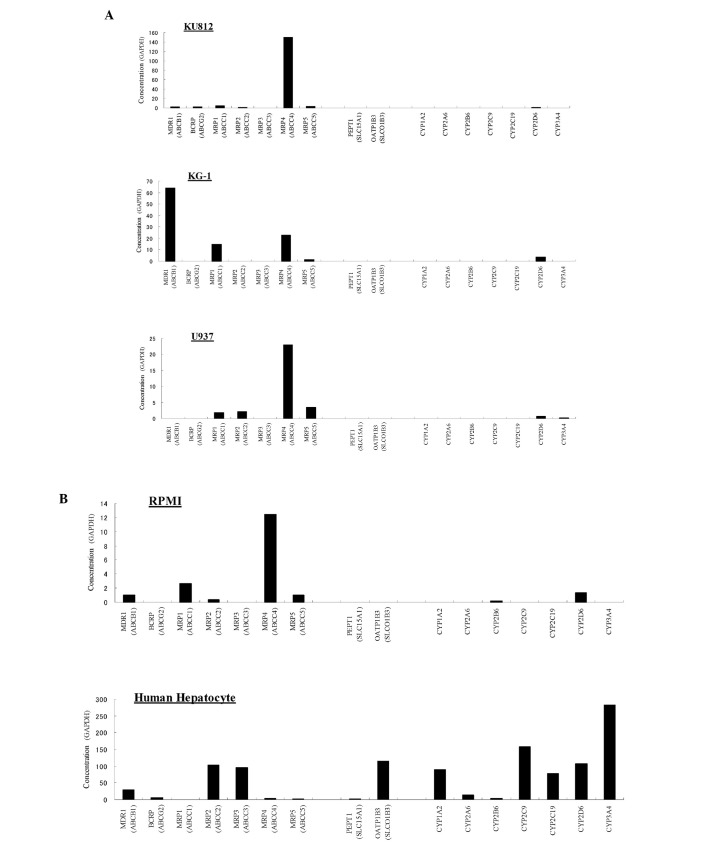

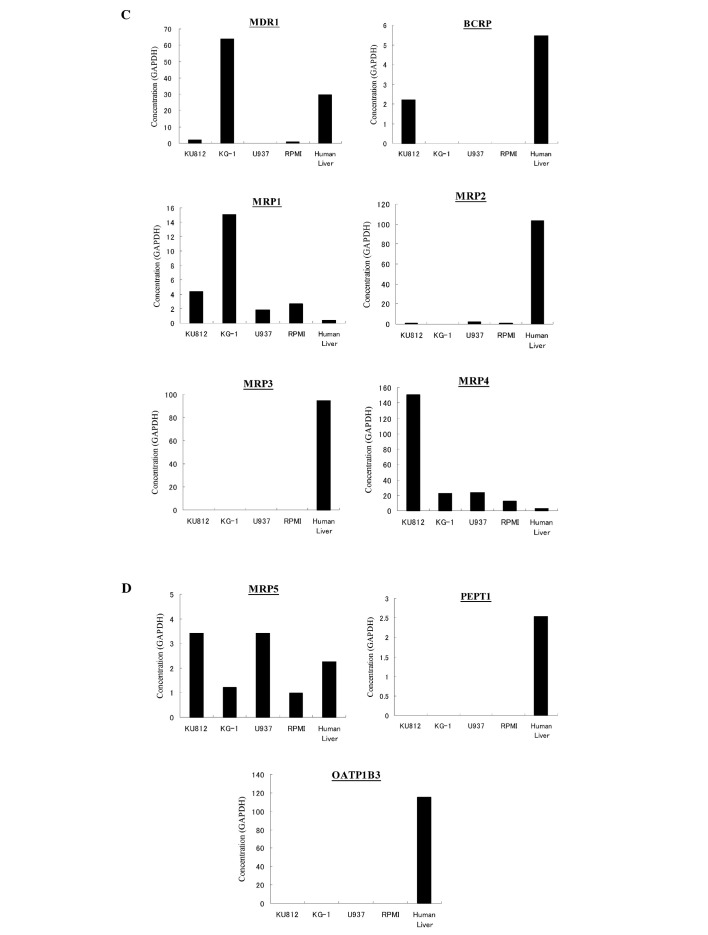
Quantitative RT-PCR analysis of the mRNA expression for typical human drug transporters and CYPs in human blood cancer cell lines and hepatocytes. The data of two independent analyses for each sample and parameter were averaged. The copy numbers are presented as the number of transcripts per 10^3^ copies of GAPDH. (A and B) Findings for each cell type. KU812; chronic myelocytic leukemia cell line, KG-1; acute myelocytic leukemia cell line, U937; lymphoma cell line, RPMI 1788; blood cell line derived from a healthy subject. §(C and D) Findings for each transporter type. KU812; chronic myelocytic leukemia cell line, KG-1; acute myelocytic leukemia cell line, U937; lymphoma cell line, RPMI 1788; blood cell line derived from a healthy subject.

The mRNA expression levels of typical ABC transporters (MDR1, BCRP and MRP1/2/3/4/5) and of SLC transporters (PEPT1 and OATP1B3) in human blood cancer cell lines were analyzed by RT-PCR. The mRNA levels of major human CYPs (CYP1A2, CYP2A6, CYP2B6, CYP2C9, CYP2C19, CYP2D6 and CYP3A4) were measured as the reference data. The mRNA expression levels of the transporters and the CYPs in human hepatocytes were also measured as a control. As a result, the expression level of MRP4 was extremely high (151 copies of transcript/10^3^ copies of GAPDH) by comparison with those of other transporters in the chronic myelocytic leukemia cell line KU812 (Fig. 1A and C). The numbers of transcripts for the other transporters in KU812 were below 4 copies/10^3^ GAPDH. In the acute myelocytic leukemia cell line KG-1, MDR1 expression level was the highest (64 copies/10^3^ GAPDH), followed by those of MRP4 (23 copies/10^3^ GAPDH) and MRP1 (15 copies/10^3^ GAPDH; Fig. 1A and C). Additionally, MRP4 expression level was the highest in the lymphoma cell line U937 (23 copies/10^3^ GAPDH) and the blood cell line derived from a healthy subject RPMI 1788 (12 copies/10^3^ GAPDH; Fig. 1A–C). Relatively low expression levels of MRP1/2/5 in the U937 cell line, and of MDR1, MRP1/2/5 in the RPMI 1788 cells were observed (Fig. 1A–D). The relative mRNA expression levels of the typical human transporters and human CYPs in the normal human hepatocytes were almost the same as those previously reported (19–21). Neither of the two major human SLC transporters (PEPT1 and OATP1B3) was detected in any of the blood cell line in the present study (Fig. 1A, B and D). With regard to the human CYPs, low expression levels of CYP2B6 in U937 and RPMI 1788, and of CYP2D6 in KU812, KG-1, U937 and RPMI 1788 were observed (Fig. 1A and B).

### Inhibitory effects of MK-571 on MTX distribution in rats

**Figure 2:**
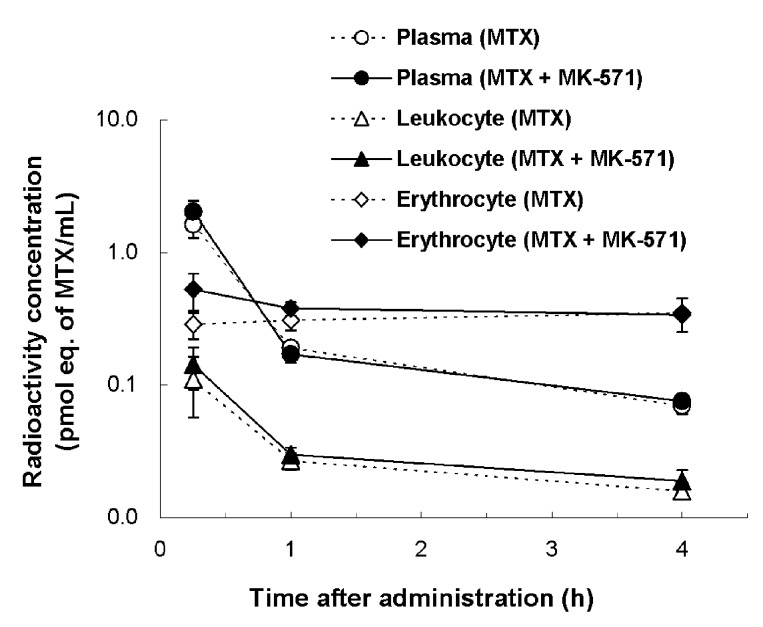
Radioactivity concentrations of plasma, leukocytes and erythrocytes after i.v. bolus administration of [^3^H]MTX alone or concomitant administration of MTX and MK-571 to rats. [^3^H]MTX (2.38 μg/kg) was administered i.v. as a bolus into the tail vein of two groups of rats. Animals in one group received [^3^H]MTX only; the other group was concomitantly administered i.v. MTX and MK-571 (1 mg/kg). Each point represents the mean ± SD of three animals.

**Figure 3:**
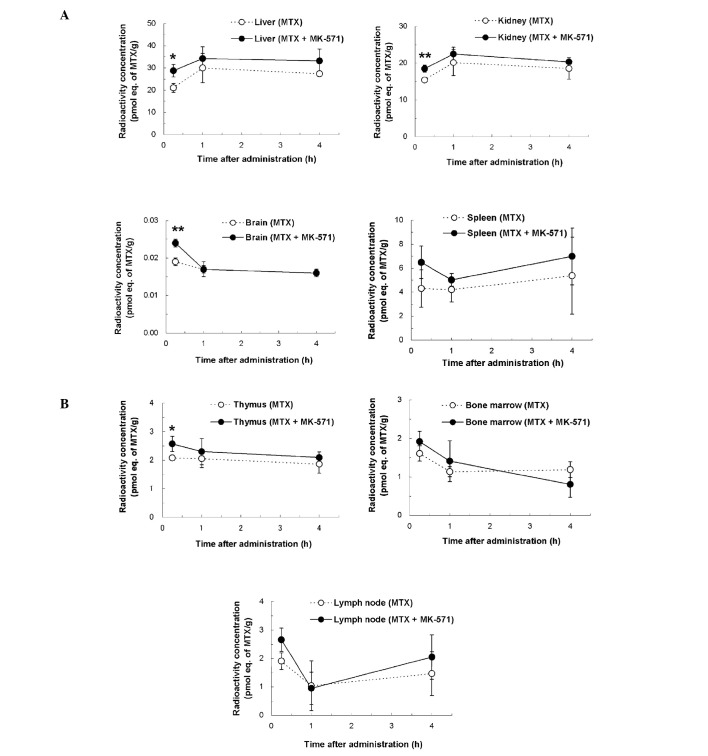
Tissue concentrations of radioactivity after i.v. bolus administration of [^3^H]MTX alone or concomitant administration of MTX and MK-571 to rats. Radioactivity concentrations in the liver, kidneys, brain, spleen, thymus, bone marrow and lymph node between the two administration groups were compared. Each point represents the mean ± SD of three animals. Significantly higher values in the concomitant administration of MTX and MK-571 (^*^P<0.05, ^**^P<0.01) by Student’s t-test. (A) Liver, kidneys, brain and spleen; (B) thymus, bone marrow and lymph node.

**Figure 4:**
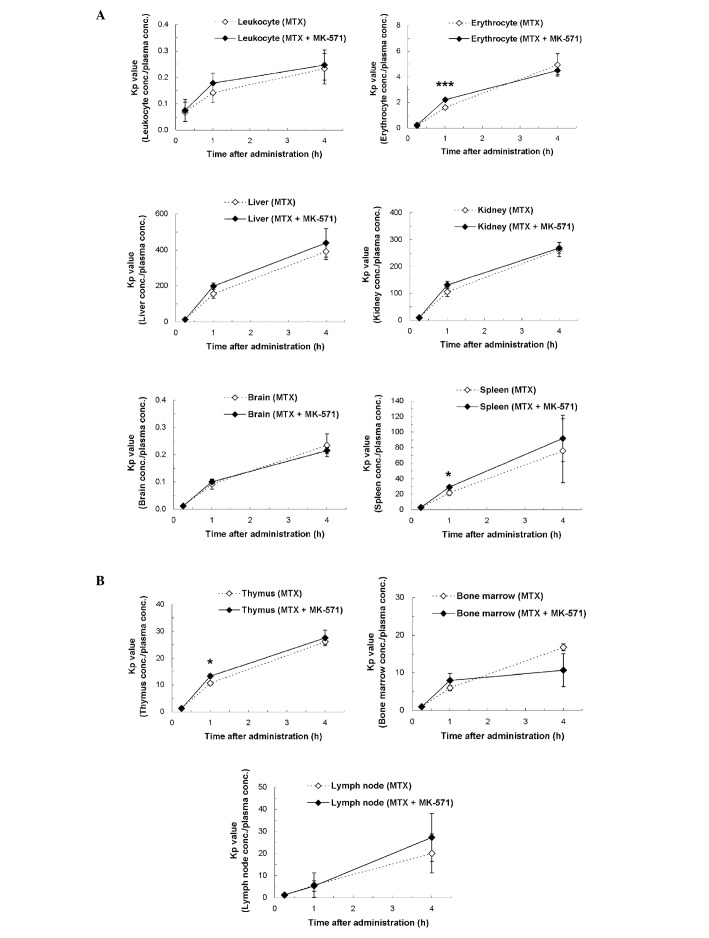
Kp values of radioactivity after i.v. bolus administration of [^3^H]MTX alone or concomitant administration of MTX and MK-571 to rats. Kp values (tissue concentration/plasma concentration) of leukocytes, erythrocytes, the liver, kidneys, brain and spleen, thymus, bone marrow and lymph node between the two administration groups were compared. Each point represents the mean ± SD of three animals. Significantly higher values in the concomitant administration of MTX and MK-571 (^*^P<0.05, ^***^P<0.001) by Student’s t-test. (A) Leukocytes, erythrocytes, liver, kidneys, brain and spleen; (B) thymus, bone marrow and lymph node.

Following the [^3^H]MTX i.v. bolus administration to rats, no significant difference in the plasma, leukocyte and erythrocyte concentrations was observed between the MTX alone and the MTX and MK-571 concomitant groups (Fig. 2). By contrast, a slight difference was observed in other tissues. The MTX concentrations in the liver, kidneys, brain and thymus at 15 min after i.v. administration in the concomitant group revealed, respectively, 1.4 (P<0.05), 1.2 (P<0.01), 1.3 (P<0.01) and 1.2 times (P<0.05) higher values compared with those of the MTX alone group (Fig. 3). The Kp values (tissue concentration/plasma concentration) of the concomitant group showed slightly higher values compared with those of the MTX alone group at 15 min after administration in erythrocytes (1.4 times, P<0.001), spleen (1.3 times, P<0.05) and thymus (1.2 times, P<0.05), respectively (Fig. 4). With regard to the distribution ratios to blood cells, no significant difference was observed between the two groups. At each time point, the mean values of the distribution ratios to blood cells in the MTX alone and the concomitant group were respectively 21.9±3.7 and 24.4±4.6% (15 min after administration), 63.7±6.6 and 67.2±1.8% (1 h) and 83.5±0.5 and 82.6±1.4% (4 h).

## Discussion

We set out to determine whether MRP4 is expressed and functions as an efflux transporter in blood cancer cells. Although direct involvement of the MRP4 function in blood cancer cells could not be evaluated in this study, the results revealed excessive expression of MRP4 in the chronic myelocytic leukemia cell line, KU812. Additionally, MRP4 expression in the normal blood cell line derived from a healthy subject, RPMI 1788, was relatively high. The phenomena associated with rodent MRP4 function have recently been suggested in normal erythrocytes of rats and in normal tissues (e.g. spleen and thymus) of mice (11,12). At present, there is insufficient data for the expression and the function for MRP4 linking cancer cells with normal blood cells/tissues. However, due to the possibility that a slight efflux function of MRP4 was suggested in normal blood cells/tissues of rats in the present study, the possibility of a functional contribution of MRP4 particularly in cancer cells with high MRP4 expression, e.g. KU812 was suggested.

To demonstrate the efficacy of anticancer drugs, direct contact between the target cancer cells and anticancer drugs at an effective drug concentration is usually required. For anti-blood cancer drugs, appropriate delivery of drugs to cancer cells (including atypical leukocytes and lymphocytes) that circulate through blood systemically, or bone marrow cells is considered to become a trigger of drug efficacy. A recent study (22) suggested that OCT1 mediates the uptake of selected anticancer drugs into lymphoma cells. It has been revealed that PEPT1 and OATP1B3 are abundantly expressed as uptake transporters in solid cancer cells. As no expression of PEPT1 and OATP1B3 was detected in blood cancer cell lines in the present study, a difference in the expression patterns of the influx transporters between solid cancer cells and blood cancer cells was suggested.

As for the efflux transporters, ABC transporters are considered to be one of the main causes of multidrug resistance to anticancer drugs, and the involvement of MDR1, BCRP and MRP1 is mainly reported at present as a drug-resistant mechanism in cancer cells (3,20). Strategies in the clinical setting for overcoming drug resistance caused by multidrug resistance proteins have been suggested (23). One of the proposed strategies is the use of agents that inhibit multidrug resistance proteins. If the expression levels of objective transporters are significantly different between cancer cells and normal cells, a selective approach may be adopted without unwanted adverse phenomena. For instance, the susceptibility of cancer cells to anticancer drugs would be expected through the use of specific uptake and/or efflux inhibition via the corresponding transporters. A previous study has revealed that the level of MRP4 expression in resistant strains of hepatocellular cancer is relatively high (24). Taking these findings together with the result that MRP4 expression in KU812 was extremely high by comparison with those of other typical ABC transporters in the present study, it was suggested that MRP4 may partly contribute to the multidrug resistance properties of cancer cells, especially for anticancer drugs that show a relatively high affinity to MRP4, e.g. MTX (10).

MTX is currently used as a therapeutic drug for acute leukemia and chronic myelogenous leukemia. As MTX is a typical substrate of MRP transporters, efflux transport of MTX via MRP transporters expressed in blood cells and tissues may be functional to a certain extent. In the case of concomitant administration of MTX and MK-571, a specific inhibitor of MRP transporters, an increase in the intracellular concentration of MTX may occur. Although no large difference was observed between the MTX alone and the MTX and MK-571 concomitant groups in the present *in vivo* study using rats, the Kp values of the concomitant group showed significantly high values (P<0.05) compared with those of the MTX alone group in erythrocytes, spleen and thymus samples. For the other tissues, slightly higher concentrations in the concomitant group were also observed. As the involvement of MRP transporters in MTX concentrations in blood cells and certain tissues was examined using normal animals in the present *in vivo* study, a more notable inhibitory effect of MK-571 would be expected in certain types of cancer cells (e.g. KU812) in which the MRP4 expression is abundant. Referring to the results that the unbound fraction of plasma MK-571 in rats was <0.5% (15) following single i.v. bolus administration, the plasma unbound concentration of MK-571 in the present study was presumed to be 0.008 nM (0.16 nM as the total concentration) at most as the concentration at 15 min after administration. MK-571 is recognized as a specific inhibitor of MRP transporters, including MRP1 (25), MRP2 (26,27) and MRP4 (28). As the reported Ki values of MK-571 for these transporters were 0.6 μM (MRP1), 13.1 to 26.4 μM (MRP2) and 10 μM (IC_50_; MRP4), the exposure concentration of MK-571 in the present study may be too low to show sufficient inhibitory effects. Although increasing the dosing concentration of MK-571 in the *in vivo* study is restricted by its solubility, higher exposure of MK-571 in blood cells and tissues may lead to an increase in the intracellular concentration of MTX caused by MRP4 inhibition.

In the development of anticancer drugs for blood cancer, KU812, KG-1 and U937 cell lines are generally used in preclinical pharmacology studies as mice xenograft models. Accordingly, the influence of MRP4, which showed high expression in these blood cancer cell lines, cannot be disregarded since the contribution ratio of transporters is usually prescribed by affinity to objective drugs and the expression amount in the target locus. Numerous studies on MRP4 function in the liver, kidneys and brain have been performed (29–31). In addition, recent reports have indicated the efflux function of MRP4 in the spleen and thymus (12). Furthermore, the drug interaction between valproic acid and carbapenem antibiotics in the clinical setting is reported to be partly explained by MRP4, i.e. the inhibition of efflux transport of valproic acid from erythrocytes by the MRP4 inhibitor, e.g. panipenem (11,32). Through further examination of the function of MRP4 and its tissue localization, avoidance of drug-drug interaction in the clinical setting, and the enhancement of the potency of objective drugs may be expected.
